# Sex-dependent effects of a gestational ketogenic diet on offspring birth and lifespan

**DOI:** 10.1371/journal.pone.0328455

**Published:** 2025-07-17

**Authors:** Sarah M. Zala, Renata Santos, Eva Strasser, Alice Schadde, Sarah Kugler, Verena Strauss, Anna Kübber-Heiss, Diana Zala

**Affiliations:** 1 Konrad Lorenz Institute of Ethology, University of Veterinary Medicine, Vienna, Austria; 2 Université Paris Cité, Institute of Psychiatry and Neuroscience of Paris (IPNP), INSERM U1266, Dynamics of Neuronal Structure in Health and Disease, Paris, France; 3 Institut des Sciences Biologiques, CNRS, 16 rue Pierre et Marie Curie, Paris, France; 4 Research Institute of Wildlife Ecology, University of Veterinary Medicine, Vienna, Austria; Universidade do Estado do Rio de Janeiro, BRAZIL

## Abstract

Low-carbohydrate, high-fat ketogenic diets (KDs) are used for treating drug-resistant epilepsy, and other potential benefits, such as treating neurological disorders, metabolic syndrome, and cancer are being explored. In addition to these and other medical applications, KDs have also become popular for rapid weight-loss and enhancing athletic performance. However, the potential negative effects of exposing developing offspring to KDs during pregnancy (gestational KD) are poorly understood, and especially the long-term health consequences. In this study, we investigated the effects of a partial gestational KD administered during the second half of pregnancy and assessed the consequences on the offspring over their entire lifespan. We found that, compared to controls, a gestational KD significantly reduced dams’ litter size and litter mass and altered the litter sex ratio, reducing the proportion of female offspring, which also had lower body mass early in their life. In contrast, male offspring exposed to a gestational KD suffered a significantly reduced lifespan and a late-onset increase in body mass. We found no evidence that a KD diet influenced some adult offspring behaviors (locomotion, anxiety, depression, circadian rhythms, food and water consumption) or reproductive success. Our results with laboratory mice may not translate to human health, but nevertheless, they should raise concerns that even a partial maternal KD during pregnancy may have detrimental effects on offspring health and longevity.

## Introduction

Ketogenic diets (KD) have been used for over a century to treat drug-resistant epilepsy and they are currently being investigated for a variety of potential therapeutic benefits, including treating neurodegenerative diseases, psychiatric disorders, metabolic syndrome, and cancer [1–4]. Recently, KDs have gained popularity for weight loss and enhancing athletic performance, despite the limited understanding of their physiological and long-term health consequences in humans [5]. KDs, characterized by low carbohydrate, moderate protein, and high fat intake, induce a metabolic switch known as ketosis, in which ketone bodies replace glucose as the primary energy source [6,7]. This metabolic shift mimics a physiological state that normally occurs during fasting, resulting in elevated serum ketones while maintaining moderate to normal blood glucose and physiological pH levels [8]. Ketone bodies — acetoacetate, β-hydroxybutyric acid (BHB), and acetone — are products of fatty acid degradation in the liver. Acetoacetate is the primary ketone produced, which is subsequently converted into BHB and volatile acetone before being released into the bloodstream. Increased BHB concentration in the blood is thus used as a marker of ketosis [9]. In target tissues, acetoacetate and BHB are efficiently metabolized into acetyl-CoA within mitochondria, fueling the tricarboxylic acid cycle and ATP synthesis [7]. However, this metabolic shift, with its drastic reduction in glycolysis, may affect tissues that rely on glycolytic ATP for specific functions, such as neurons in the brain [9–12]. Besides its metabolic role, BHB acts as a direct and indirect signaling molecule, for instance in epigenetic regulation, through direct histone β-hydroxybutyrylation and also indirectly by modifying histone acetylation and methylation, as well as by regulating catabolism [13,14]. Overall, a KD induces a metabolic reprogramming that is accompanied by pleiotropic physiological effects on the organism. However, there is a lack of reliable information for the general public regarding the potential benefits and risks of KDs during pregnancy. For example, web searches yield conflicting results, with some sources asserting that a gestational KD is dangerous, while others claim that there is no evidence of any risks or that it is completely safe. Additionally, there are a significant number of KD cookbooks available for pregnant women. The problem is that prolonged glucose restriction might cause lasting and potentially irreversible alterations, particularly during *in utero* development.

The impact of a gestational KD on offspring development, health, and survival remains largely unexplored and so the results can be briefly summarized. One study reported that sons of epileptic mothers treated with a moderate KD during pregnancy displayed normal development at birth [5,15]. In contrast, studies on gestational diabetes mellitus revealed an inverse correlation between maternal gestational ketone levels and the intelligence quotient of offspring [9, 16]. Studies in rodents *in vitro* show that a gestational KD during the entire gestation period affects offspring as early as embryonic pre-implantation stages [17–20] and induces *in vivo* postnatal developmental delays, structural brain differences, and behavioral abnormalities that persist into adulthood [21–23]. Conversely, other research found that a gestational KD exposure reduced depression- and anxiety-like behaviors and increased sociability, without altering oxytocin expression [24,25]. Lactation is also a critical period affected by KDs, as adverse effects have been observed in both lactating rats and their offspring [21]. Additionally, a severe case of starvation ketoacidosis in a breastfeeding mother has been documented [26]. These findings in humans and rodents underscore the complexity of gestational KDs, emphasizing the need for further research in this area.

In the present study, we investigated the effects of a gestational KD limited to the organogenesis period (late gestation) and assessed its impact across the entire lifespan of the offspring. Our findings reveal that even a partial, 10-day maternal metabolic shift to ketosis can have sex-specific detrimental effects during critical life stages. Female offspring were adversely affected at birth, while male offspring had a reduced longevity and an increased body mass late in life.

## Results

### Induction of a partial gestational ketosis with a 10-day ketogenic diet

**Fig 1:**
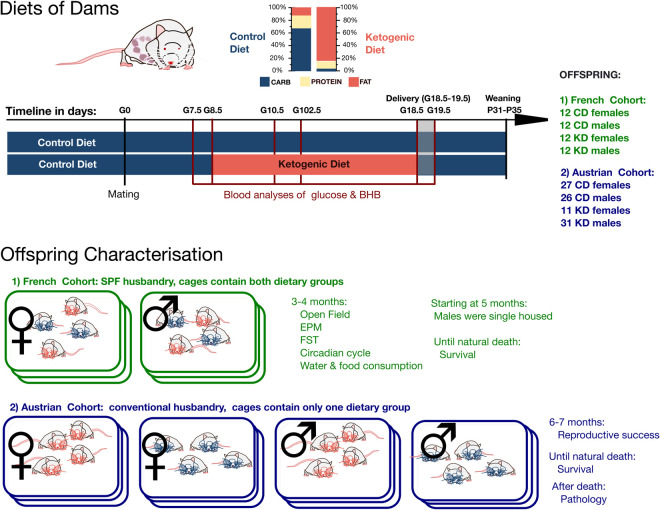
Experimental timeline. In the upper portion, timeline and manipulation of maternal diet until weaning of the offspring. Sample sizes depict the offspring number in the two cohorts. In the lower portion, the husbandry conditions illustrated per cage (squares around the mice) and offspring age during the behavioral assays. Green color: French cohort; Blue color: Austrian cohort. Abbreviations: CD = control diet, KD = ketogenic diet, BHB = β-hydroxybutyric acid, EPM = elevated plus maze, FST = forced swim test, SPF = specific pathogen free.

Previous studies showed that a prolonged KD exposure before, during, and after gestation reduced fertility, delayed embryonic development, and induced organ-specific changes in the progeny [20,27]. Additionally, *in vitro* studies reported that physiological concentrations of ketones induced epigenetic modifications in blastocysts, impaired implantation, and caused female-specific developmental perturbations [18,19]. In the present study, to investigate the effects of a gestational ketosis specifically at late embryogenesis, a KD administration was initiated at gestational day (G)8.5, when organogenesis begins, after the fetal neural plate and heart tube had already formed (see Fig 1 for the timeline of the experiment, and Table 1 and S1 Table for the dietary compositions). The diet was terminated at G18.5 to prevent potential adverse effects during lactation. The KD used in this study comprised 5% carbohydrates, 11% protein, and 84% fat (Table 1). This carbohydrate proportion closely mirrors human KD formulations but is significantly higher than the ultra-low carbohydrate content (<1%) commonly used in murine studies [24,25]. This partial and mild dietary intervention allowed us to focus on the effects of a gestational ketosis on organogenesis while minimizing the broader disruptions associated with prolonged or more extreme dietary regimens.

**Table 1:** Diet composition. CD = control diet, KD = ketogenic diet, SD = standard diet.

Diet	Fat (%kJ/kg)	Protein (%kJ/kg)	Carbohydrates (%kJ/kg)
CD (C1000)	13	20	67
KD (C1086)	84	11	5
SD (France) SAFE A04	8	19	72
SD (Austria) V1534, Ssniff	9	33	58

**Fig 2:**
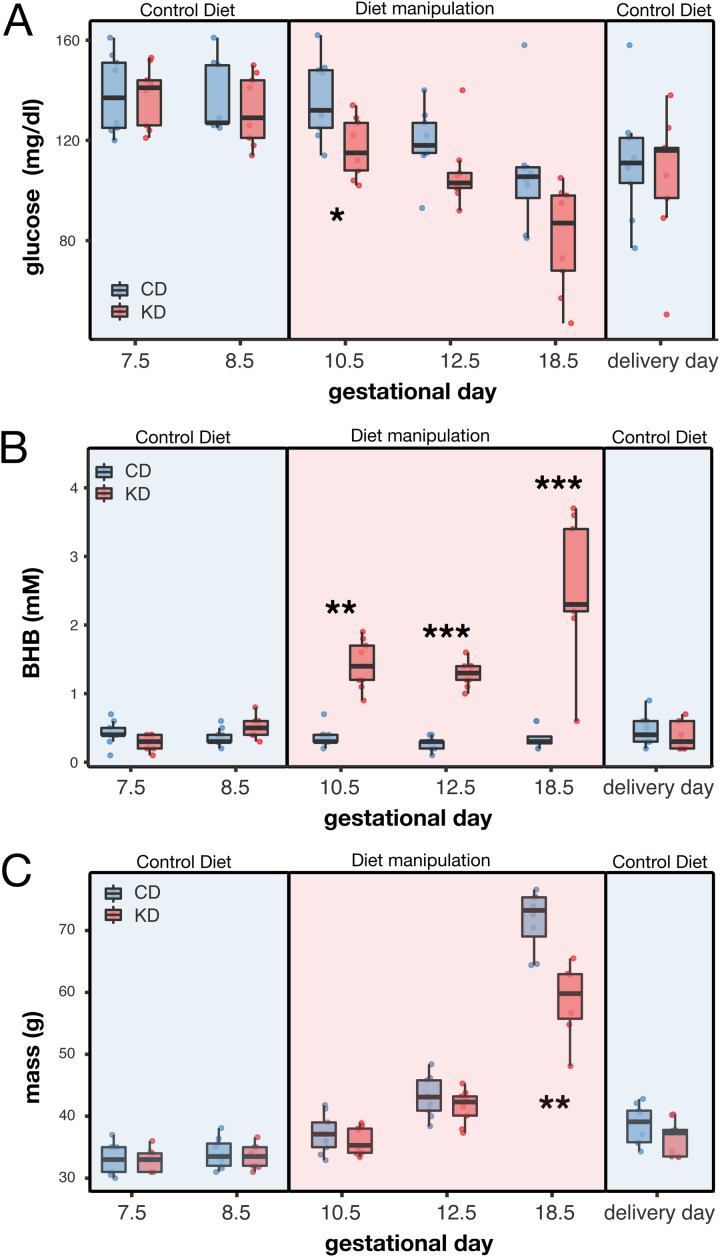
Effects of a partial KD in dams’ glucose and ketone levels and body mass during gestation. (A-C) Box plots showing the dynamics of (A) blood glucose, (B) BHB, and (C) body mass in dams fed either a control diet or ketogenic diet (**p < 0.01, ***p < 0.001). Box plots include all values with outliers, median, individual values, first and last quartiles, whiskers drawn within the 1.5 interquartile range value.

Blood glucose and BHB levels were monitored throughout the gestation, with measurements taken pre-diet (G7.5 and G8.5), during the diet (G10.5, G12.5, and G18.5), and post-diet (G19.5, after reintroduction of the control diet). As expected, during the KD period the KD group exhibited a moderate reduction in blood glucose levels (ANOVA with repeated measurements: n = 17, time: F = 18.50, p < 0.00001; time*diet: F = 2.07, p = 0.008). This reduction was statistically significant at G10.5 (T-test: n = 8, T = 0.79, p = 0.016; Fig 2A). In contrast, BHB levels significantly increased during the KD period, confirming a state of ketosis (ANOVA with repeated measurements: time: n = 16, F = 12.396, p < 0.0001; time*diet: n = 16, F = 10.868, p < 0.0001; post-hoc test: p < 0.001 at G10.5, G12.5, and G18.5; Fig 2B). These results confirm that a gestational ketosis was successfully induced. As expected, the body mass of pregnant females in both groups increased throughout gestation and decreased after delivery. However, at G18.5 the KD mice showed a significantly lower body mass compared to the CD group (ANOVA with repeated measurements: n = 16, time: F = 398.24, p < 0.00001; time*diet: F = 77.40, p < 0.00001; post-hoc test: p = 0.003 at G18.5; Fig 2C). This difference resolved post-delivery.

In summary, our partial KD effectively induced ketosis, maintained physiological glucose levels, although reduced compared to a control diet, and resulted in a transient reduction in maternal body mass during late gestation.

### A partial gestational KD influenced litter size, offspring sex ratio, and pup body mass

**Fig 3:**
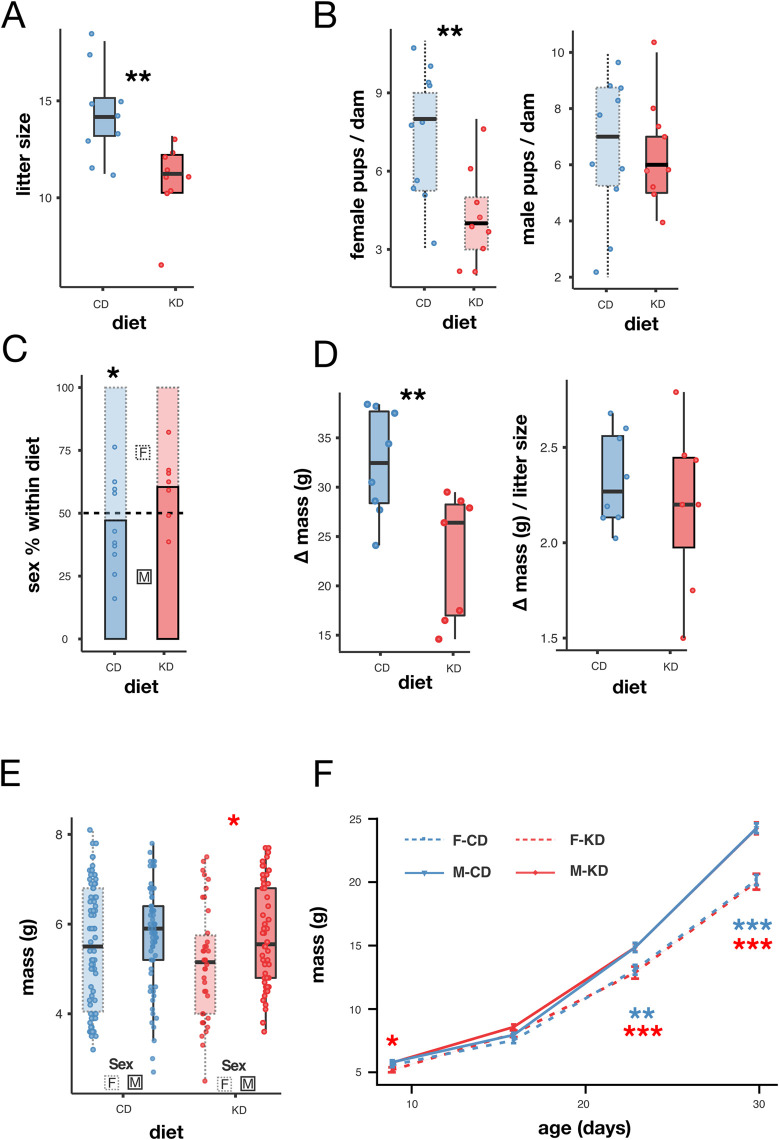
A partial gestational KD impacted female, but not male, pups. (A) Litter size by gestational diet. (B) Number of female (left) and male (right) pups per litter by diet. (C) Offspring sex ratio by diet. (D) Maternal body mass difference between G18.5 and post-delivery (G18.5-G19.5) by diet (left) and estimated mean pup mass calculated by dividing the maternal mass difference by the litter size (right). (E) Pup body mass at P9 by diet and sex. (F) Dynamics of pup body mass during the first month by diet and sex (mean±SEM). Box plots include all values with outliers, median, individual values, first and last quartiles, whiskers drawn within the 1.5 interquartile range value. Statistics A, B, D, and E: Student’s T-test; C: χ² test, F: repeated measures ANOVA. Significance levels: *p < 0.05, **p < 0.01, ***p < 0.001.

Our gestational KD had a striking effect on the litter size and sex ratio of the offspring at postnatal days 4–9 (P4-P9). Litter size was significantly reduced in the KD group compared to the CD offspring (independent samples T-test: n = 9 for KD and n = 10 for CD, T = 3.29, p = 0.005; Fig 3A). Notably, one female in the KD group failed to deliver, and this animal was conservatively excluded from the analysis. Unexpectedly, the number of female pups per litter was significantly reduced in the KD group, while the number of male pups remained unchanged (independent samples T-test: n = 9 for KD and n = 10 for CD; females: T = 3.04, p = 0.007; males: T = 0.15, p = 0.885; Fig 3B). The offspring sex ratio in the CD group adhered to a Mendelian distribution (74 females and 66 males; 52.9% females; Fig 3C). However, in the KD group, females comprised only 39.6% of the offspring (38 females and 58 males; χ² test: p = 0.045). These findings suggest that female embryos were more vulnerable to gestational ketosis, potentially leading to increased embryonic lethality.

Maternal body mass pre-delivery (G18.5) and post-delivery (G19.5) was compared to estimate the total delivery mass as a proxy for litter mass. A significant reduction in this mass was observed in the KD group compared to the CD group (T-test: n = 7 for KD and n = 8 for CD, T = 3.06, p = 0.009; Fig 3D). When normalized by litter size, the average mass per pup showed a smaller and non-significant reduction in the KD group (independent samples T-test: n = 7 for KD and n = 8 for CD, T = 0.78, p = 0.450; Fig 3D). These results contain mean values for both males and females. To further evaluate offspring growth, pup body mass was monitored weekly from postnatal day nine (P9) to weaning. While male and female pups in the CD group had comparable body masses at P9 (independent samples T-test: T = 1.033, p = 0.302; n = 65 for males and n = 74 for females; Fig 3E), female pups in the KD group were significantly lighter than KD males at this stage (independent samples T-test: T = 2.32, p = 0.023; n = 58 for males and n = 38 for females; Fig 3E). This finding suggests that female pups from the KD dams were already smaller at birth and that this difference persisted for at least nine days, despite the fact that during postnatal lactation the mothers were no longer in ketosis. At P16 female body mass in the KD group did not differ from male body mass (T = 1.81, p = 0.074; Fig 3E). However, starting at P23 a significant sexual dimorphism in body mass was evident in both dietary groups, with males consistently weighing more than females, as expected (ANOVA with repeated measures: age p < 0.0001, age*sex p < 0.0001, age*diet p = 0.012, Post Hoc Test with Holm correction at P23 and P31, sex: p < 0.0001, Fig 3F, n = 65 for CD females; n = 37 for KD females; n = 65 for CD males and n = 58 for KD males).

In summary, a partial gestational ketosis had detrimental effects on female offspring by reducing their number at birth and their initial body mass, whereas male offspring appeared unaffected.

### A partial gestational KD had no lasting impact on offspring metabolic profile

**Fig 4:**
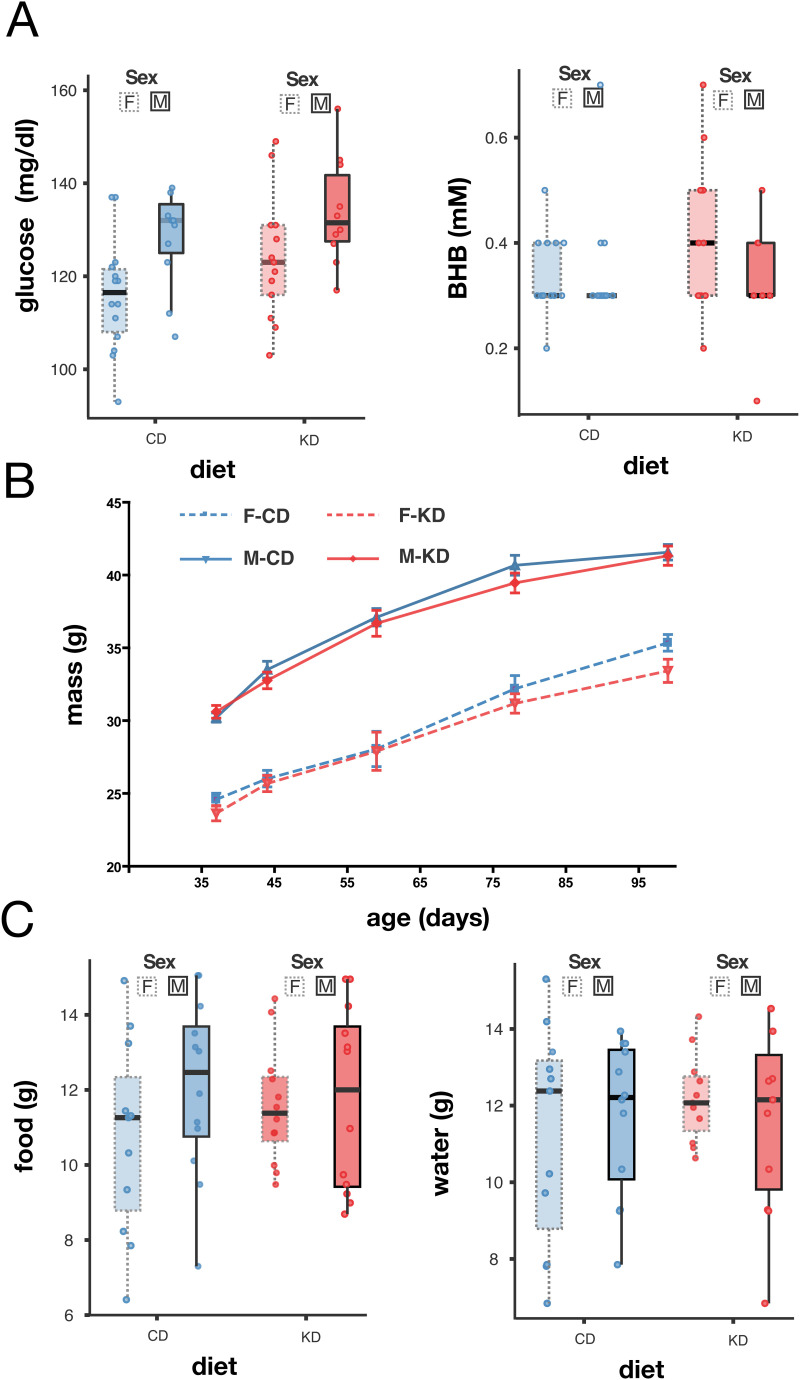
A partial gestational KD did not affect metabolism, body mass, nor food and water intake. (A) Blood glucose (right) and blood BHB levels (left) in adult offspring by diet and sex. (B) Body mass dynamics across time by diet and sex. (C) Total food (left) and water consumed over 78 h. Box plots include all values with outliers, median, individual values, first and last quartiles, whiskers drawn within the 1.5 interquartile range value. Statistics A-C: ANOVA, p > 0.05.

To avoid potential confounding effects of extreme litter sizes [28,29], two outliers from the CD group (litter sizes of 16 and 17 pups) were excluded from further analysis. Glucose and BHB levels were measured in adult offspring at four months of age. All offspring displayed glucose levels within the normal glycemic range and low BHB levels, as expected, indicating no lasting metabolic alterations from the gestational KD exposure (ANOVA: sex*diet, for glucose p = 0.552, F = 0.353; for BHB p = 0.668; F = 0.186; n = 18 and n = 17; Fig 4A). In addition, there was no difference in body mass (ANOVA between subjects effects: sex*diet: n = 19–39, p = 0.734, F = 0.115, df = 228; Fig 4B). Finally, no differences were observed in food and water consumption (ANOVA: sex*diet, food: p = 0383, F = 0.383; water: p = 0.965, F = 0.002 n = 10–12; Fig 4C). These findings suggest that a partial gestational KD exposure did not result in lasting metabolic effects on adult offspring.

### A partial gestational KD had no lasting impact on offspring anxiety- and depressive-like behavior

**Fig 5:**
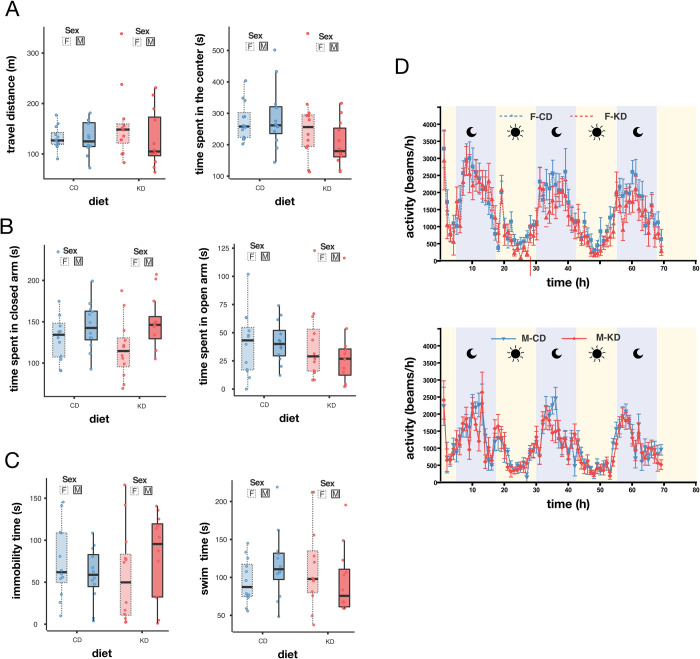
A partial gestational KD did not affect adult offspring spontaneous locomotion, susceptibility to anxiety and depression, nor circadian rhythms. (A) Total distance traveled during 1 hour in the open field test (left) and time spent in the center (right) by diet and sex. (B) Time spent in the closed arm (left) and the open arm (right) during a 5-minute elevated plus maze assay. (C) Time spent immobile (left) or actively swimming (right) during a 5-minute forced swim test. (D) Locomotor activity in the light and dark cycles. Box plots include all values with outliers, median, individual values, first and last quartiles, whiskers drawn within the 1.5 interquartile range value. Line graphs (D) shows mean±SEM. Statistics A-C: Anova p > 0.05.

In a previous study, Sussmann *et al*. [24] reported that a full gestational KD in CD-1 mice reduced susceptibility to anxiety and depression, as assessed using the open field test (OFT), elevated plus maze (EPM), and forced swim test (FST), compared to controls on a standard diet. In the present study, we evaluated spontaneous activity and found no differences in the total distance traveled in the OFT (ANOVA: sex*diet p = 0.337, F = 0.944, n = 12) or the time spent in the center of the arena (ANOVA: sex*diet p = 0.205, F = 1.658, n = 12; Fig 5A). Similarly, the EPM test revealed no significant differences in the time spent in the open or closed arms (ANOVA: sex*diet p = 0.872, F = 0.026, n = 12; Fig 5B). Additionally, depression-like behavior assessed via the FST [30] also showed no significant differences between sex and diet groups (immobility, Kruskal-Wallis: χ² = 2.1828, p = 0.535, n = 12; Fig 5C left; swim time, Kruskal-Wallis: χ² = 2.028, p = 0.567, n = 12; Fig 5C right).

### A partial gestational KD had no lasting impact on circadian rhythms

Dietary interventions, such as high-fat diets and caloric restriction, can disrupt the oscillatory expression of genes, thereby altering circadian rhythms [31,32]. For example, KDs increase ketone body production, mimicking a fasting state and causing shifts in circadian phases. The establishment of circadian rhythms begins during late embryonic development and is influenced by maternal factors [33–35]. To investigate whether maternal ketosis induced circadian changes in our model, we evaluated night-day rhythms. Our results showed no significant differences between gestational KD and control animals (Fig 5D).

In conclusion, a 10-day gestational ketogenic diet caused no significant behavioral abnormalities related to anxiety, depression, spontaneous activity or circadian rhythms in the adult offspring.

### A partial gestational KD had no lasting impact on offspring reproductive success

**Fig 6:**
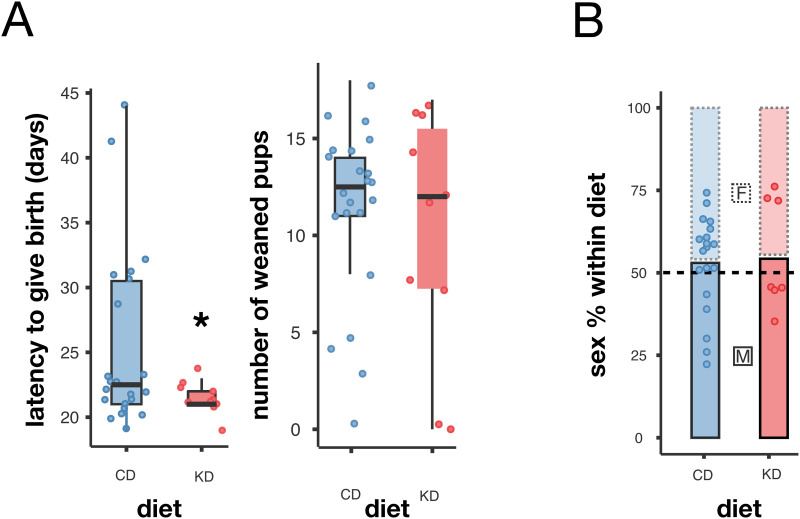
A partial gestational ketogenic diet had no lasting impact on offspring’s reproductive success. (A) Latency to give birth (left) and number of weaned pups (right) by diet. (B) Offspring sex ratio by diet. A: T-test, p < 0.05; B: χ² test, p > 0.05. Box plots include all values with outliers, median, individual values, first and last quartiles, whiskers drawn within the 1.5 interquartile range value.

To measure offspring fertility and reproductive success, we bred 22 gestational CD and 10 gestational KD pairs in the Austrian cohort. All 32 females successfully gave birth; however, two gestational KD females and one CD female cannibalized their offspring post-delivery. The mean (±s.d.) of latency to give birth differed significantly between the groups, with the gestational KD group delivering at 21.5 ± 1 days and the CD group at 25.5 ± 7 days after pairing (T-test with unequal variances: T = −2.6, df = 24.3, p = 0.016; Fig 6A). Despite this difference in latency to give birth, no significant differences in reproductive success were observed when offspring were weaned (mean number of weaned offspring: KD = 10.2 ± 6, CD = 11.3 ± 5; T-test: T = 0.5, df = 30, p = 0.59). Additionally, the offspring sex ratio followed a Mendelian distribution in both groups (CD: 115 females and 133 males; KD: 46 females and 56 males; χ² test: p = 0.828; Fig 6B).

### A partial gestational ketogenic diet resulted in late-life increased body mass and shortened lifespan of male offspring

**Fig 7:**
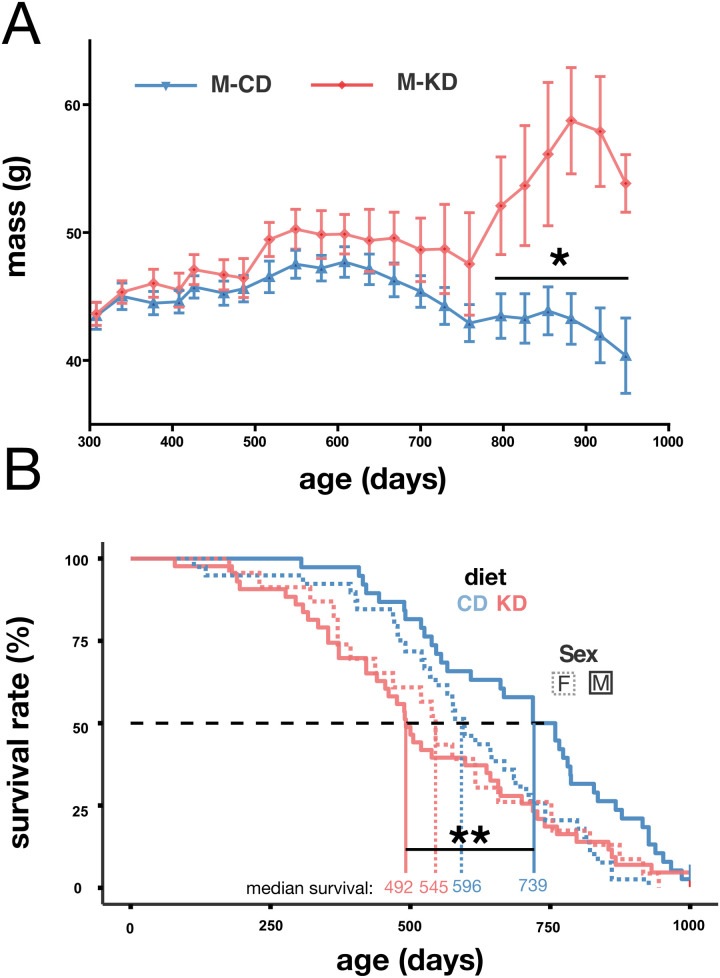
A partial gestational KD impacted male lifespan and mass during late-life. (A) Body mass dynamics across time by diet: Repeated ANOVA, p < 0.05. (B) Survival rate of the offspring by sex and diet: Log-rank test: χ² = 7.47, p = 0.006.

We found that a maternal KD during gestation resulted in reduced female offspring body mass shortly after birth (Fig 3E), though this difference resolved by P16 (Fig 3F). No further differences in female body mass were detected throughout their life until their natural death (Fig 4B and S2 Table). No differences in body mass were observed in KD male offspring until approximately 2.2 years of age (Figs 3E–3F, 4B, 7A) when a significant increase in body mass was found, which persisted until their natural death (Fig 7A).

We assessed offspring longevity in two cohorts (one in France and one in Austria) with two different husbandry systems, one specific pathogen free (SPF) and one conventional (see Materials and Methods). The long-term survival of offspring did not significantly differ between the French and the Austrian cohorts (Log-rank test: χ² = 2.14, p = 0.144), which permitted pooling these data (Fig 6B). No sex-bias was detected in the overall longevity (Log-rank test: χ² = 1.285, p = 0.257). However, median survival was significantly reduced in the KD offspring (512 days) compared to controls (661 days) (Log-rank test: χ² = 7.47, p = 0.006). Sex- and diet-specific analyses revealed that male KD offspring had a significantly reduced median survival of 247 days compared to controls (KD: 492 days; CD: 739 days; Log-rank test: χ² = 9.459, p < 0.0021, Fig 7B). In contrast, female survival was only slightly affected by the diet, with median survival differing by 51 days (KD: 545 days; CD: 596 days; Log-rank test: χ² = 0.38602, p = 0.2344).

In summary, a partial gestational ketogenic diet had lasting effects on male offspring, including a late-onset increase in body mass and a reduction in lifespan, but these effects were not found in females.

### A partial gestational ketogenic diet did not alter pathological outcomes in offspring

We performed pathological examinations on 66 mice (CD: n = 40, KD: n = 26), including 21 that were euthanized due to health issues and others found dead in their cages. The examined Austrian cohort included 21 males and five females from the KD group, and 19 males and 21 females from the CD group (Table 2). Parasitological analysis identified oxyurid infections in four mice (KD: n = 1, CD: n = 3).

**Table 2:** Offspring pathology: Type of neoplasia and level of malignancy according to diet and sex. Note that some individuals had more than one type of neoplasia. Each row shows the number (and % total) of mice with the specific disease.

		Control Diet			Ketogenic Diet		
Type of neoplasia	Level of malignancy	Females (n = 21)	Males (n = 19)	Total (n = 40)	Females (n = 5)	Males (n = 21)	Total (n = 26)
Lymphoma	Malignant	10	6	40%	2	11	50%
Pancreatic islet cell carcinoma	Malignant	2	1	7.5%	2	1	11.5%
Pulmonary carcinoma	Malignant	5	3	20%	0	3	11.5%
Hemangiosarcoma	Malignant	1	4	12.5%	0	1	4%
Uterine carcinoma	Malignant	1	0	2.5%	1	0	4%
Squamous cell carcinoma	Malignant	3	1	10%	0	0	0%
Fibrosarcoma	Malignant	0	2	5%	0	0	0%
Renal carcinoma	Malignant	0	2	5%	0	0	0%
Mammary carcinoma	Malignant	2	0	5%	0	0	0%
Hepatic adenoma	Benign	1	0	2.5%	0	4	15%
Pulmonary adenoma	Benign	0	1	2.5%	0	1	4%
Renal adenoma	Benign	0	1	2.5%	0	1	4%
Adenoma of Harderian gland	Benign	0	1	2.5%	0	1	4%
Trichoepithelioma	Benign	0	1	2.5%	0	0	0%
Sebaceous gland adenoma	Benign	1	0	2.5%	0	0	0%
Non-neoplastic	Non-neoplastic	1	3	10%	1	6	27%

One or more neoplastic lesions were observed in 73% (n = 19/26) of KD offspring, with 69% (n = 18) presenting malignant neoplasms. Among these, multicentric lymphoma was the most common (50%), followed by pancreatic islet cell carcinoma (11.5%), pulmonary carcinoma (11.5%), hemangiosarcoma (4%), and uterine carcinoma (4%). Four KD offspring also exhibited hepatic adenomas, while isolated cases of pulmonary adenoma, renal adenoma, and Harderian gland adenoma were noted. Seven KD mice died due to non-neoplastic causes, such as pneumonia or enteritis.

One or more neoplastic lesions were also very frequent in the CD offspring, affecting 90% (n = 36/40), with 85% (n = 34) developing malignant neoplasms. Multicentric lymphoma was again predominant (30%, n = 12), followed by lymphomas in the spleen or lymph nodes (10%), pancreatic islet cell carcinoma (7.5%), pulmonary carcinoma (20%), hemangiosarcoma (12.5%), squamous cell carcinoma (10%), and fewer cases of fibrosarcoma, renal carcinoma, uterine carcinoma, and mammary carcinoma. Benign neoplasms were identified in two CD mice, including pulmonary adenoma, hemangioma, and sebaceous gland adenoma. Non-neoplastic causes of death were recorded in four CD offspring.

No significant differences were found between dietary groups or sexes in the prevalence of lymphoma, lung carcinoma, or pancreatic carcinoma (Table 3). Other neoplastic lesions were rare and not statistically compared.

**Table 3:** Logistic regression table: Effects of diet and sex on diagnosed neoplastic lesions. There are no significant differences between the dietary groups and sex in the occurrence of lymphoma, lung carcinoma or pancreatic carcinoma.

Generalized Linear Model				
Lymphoma:				
Coefficients:				
	*Estimate*	*Std. Error*	*z value*	*Pr(>|z|)*
(Intercept)	−0.7732	0.4935	−1.567	0.117
Sex	0.6779	0.6592	1.028	0.304
Diet	0.8685	0.6592	1.318	0.188
Sex:Diet	−1.1787	1.2078	−0.976	0.329
Pancreatic islet cell carcinoma:				
Coefficients:				
	*Estimate*	*Std. Error*	*z value*	*Pr(>|z|)*
(Intercept)	−2.8904	1.0274	−2.813	0.0049
Sex	0.6391	1.2681	0.504	0.6143
Diet	−0.1054	1.4510	−0.073	0.9421
Sex:Diet	1.9512	1.8685	1.044	0.2964
Pulmonary carcinoma:				
Coefficients:				
	*Estimate*	*Std. Error*	*z value*	*Pr(>|z|)*
(Intercept)	−1.6740	0.6292	−2.661	0.0078
Sex	0.5108	0.8114	0.630	0.5290
Diet	−0.1178	0.8858	−0.133	0.8942
Sex:Diet	−16.2851	1769.2579	−0.009	0.9927

## Discussion

In this study, we found that maternal exposure to a KD beginning at the organogenesis stage resulted in long-lasting, sex-specific effects on offspring health and survival. Female offspring were adversely affected at birth, whereas male offspring showed reduced survival later in life. Specifically, gestational ketosis resulted in smaller litter sizes, in reduced female pup mass, and in sex-dependent female fetal loss. Interestingly, while our gestational KD did not affect males at birth, it negatively impacted aging, with KD-exposed males having a shorter lifespan.

A previous study in CD-1 mice reported that KD exposure before mating reduced fertility and litter size [27]. However, in the present study, a shorter gestational KD primarily affected female offspring at birth. Female offspring might have been affected before being born, or, alternatively, they might have been selectively cannibalized by their mothers after birth, suggesting a complex and time-dependent impact of maternal diet on fetal development. In humans, male-biased birth ratios are well-documented, with the World Health Organization (WHO) estimating an expected ratio of 103–107 boys per 100 girls (https://ourworldindata.org/gender-ratio). Although the sex ratio at conception is equal, female fetuses have a higher probability of abortion, suggesting that they are slightly more vulnerable during gestation [36]. Evidence from mice suggests that low-fat, high-carbohydrate diets favor female offspring [37,38], whereas high-fat diets promote a male bias [39]. Female placentas are more sensitive to gene regulation influenced by dietary fat content than male placentas [40,41], potentially explaining the heightened vulnerability of female fetuses to the synergistic deleterious effects of both high fat and low carbohydrates of KDs.

While KDs have been shown to benefit adult mice in reducing anxiety and depression [3, 42] and a full gestational KD has been reported to improve outcomes in adult offspring [24,25], we were unable to replicate these beneficial effects. Differences in the KD composition (5% carbohydrates in our study compared to <1% in others [24,25]) or the timing of diet initiation (our dietary manipulation began at late gestation) could explain these discrepancies. Crucially, our KD was administered prior to critical stages of brain development and after the fetal neural plate and heart tube had already formed, and pregnant females exhibited a controlled ketosis, as shown by increased ketone levels while maintaining a normal glucose homeostasis.

Several studies have shown that early development can have permanent effects in mammals [43,44] and that early development can also be a key factor influencing an individual’s lifetime reproductive success [43,45]. Although reproductive success was not affected in the offspring of our study, females of the gestational KD group had a shorter latency to give birth after male introduction compared to controls. This statistical difference, however, can be explained by the high variance in the control groups (driven by a few outliers) and the smaller sample size of the KD group. Further studies focusing on behavioral phenotypes, particularly social interactions and courtship, are needed to better characterize multiple effects of a gestational KD on social behavior.

Additionally, we observed a significant reduction in the longevity of male offspring born to KD-exposed mothers. Note that, while the offsprings’ housing conditions (SPF-free *versus* conventional mouse facilities) and standard diets in the French and Austrian animal facilities differed (both diet formulations are standard rodent facility diets), we didn’t find any difference in survival rates between the two cohorts despite such housing and diet differences. These findings highlight the robustness and reproducibility of our data across different housing and diets, a key test for reproducibility and generalizability. As CD-1 mice are known to develop spontaneous neoplasms with age [46], we investigated possible causes of reduced longevity of male offspring born to KD exposed mothers and performed pathological analyses on a subset of mice in Austria. However, even though our pathological analysis confirmed that most deceased mice had at least one neoplasm, they also showed that the gestational KD did not alter the prevalence of neoplasms. Thus, although KDs in adult mice have been shown to extend longevity and health span [47], our findings suggest that a KD during gestation causes the opposite. Interestingly, gestational exposure to high-fat diets has also been associated with positive effects on brain health and aging in both male and female offspring [48], and future studies should investigate brain development and aging, as well as the causes affecting longevity.

Together, our results highlight the complex and sex-dependent effects of a gestational KD on offspring development, survival, and health. These findings underscore the need for further research to delineate the specific mechanisms underlying these outcomes and to refine dietary recommendations during pregnancy.

### Future directions

There are some limitations in our study that should be considered. First, although we detected no differences in the adult offspring behavior and metabolism of the KD mice compared to the CD mice using four standard behavioral assays, we may have overlooked changes in other behaviors, such as social interactions, which should be investigated in future studies. Similarly, a better characterization of the metabolism by a glucose tolerance test would be required. Second, although we found differences in longevity, the reasons for the premature mortality of the KD mice are still unclear. The pathology results detected a variety of malignancies as well as benign neoplasms among the deceased mice but found no differences in the incidence of neoplasms between treatment versus control mice, despite differences in their longevity. Pathology was not conducted on all the mice that died unfortunately, and since we missed 19 mice that died before reaching 471 days of age, the pathology results are biased towards older mice. In addition, we only have data on five female offspring of the KD group and due to this small sample size, the lack of sex differences of the KD group is inconclusive. Future studies are thus needed to determine the pathological causes of early mortality. Interestingly, later in life, male offspring of the KD group showed a greater body mass than the controls. While we showed that our dietary manipulation had no lasting metabolic effects on adult KD offspring at four months of age, we cannot exclude that their metabolism may still be altered at very late stages of life. Interestingly, however, the pathology findings indicate that the heavier mass of the dissected mice was not due to obesity, as excessive fat tissue was not seen. The mass of mice can vary over time due to several reasons, such as water retention, stomach and intestinal contents, but also pathological conditions, such as neoplasms. As our pathological investigations did not measure factors such as ingesta or neoplasm mass, future studies are needed to understand the cause of this increased male body mass.

## Materials and methods

### Animals

Mice (RjOrl:SWISS, Janvier Labs) were housed in ventilated cages in a specific-pathogen-free (SPF) facility in France, with food and water provided *ad libitum*. Housing conditions were maintained at a constant temperature (23 ± 1°C) with a 12 h light:dark cycle. An additional cohort of mice (n = 95 offspring, see below) was housed in Austria under similar standard conditions, although in a conventional husbandry with open cages [49]. For survival studies, we used open cages with improved enrichment at both sites. The overall health status of the animals was checked daily by trained professionals. Animal welfare assessment criteria included monitoring general appearance and mouse behavior. Mice were euthanized according to animal welfare guidelines when a humane endpoint was reached (e.g., tumor-associated ascites, decreased response to stimuli, and lethargy).

All experiments were conducted in accordance with the Council of the European Union Directive 2010/63/EU of 22 September 2010 on the protection of animals used for scientific purposes. The study was approved by the French Ethical Committee (APAFIS#27654−202103101508446 v3) and by the Ethics and Animal Welfare Committee of the University of Veterinary Medicine, Vienna (ETK-027/02/2021) in accordance with the University’s guidelines for Good Scientific Practice.

### Dietary intervention and offspring

Upon arrival, all animals were switched to and maintained on a control diet (CD: total energy content (kJ): 13% fat, 20% protein, 67% carbohydrate; C1000 from Genestil). Mating was performed overnight using 6-week-old animals, and the presence of a sperm plug the following morning was designated as gestational day 0.5 (G0.5). Pregnant females were randomly assigned (https://www.random.org/) to either the control group or the ketogenic group, with assignment balanced based on the initial day of gestation. For 10 days, from G8.5 to G18.5, females in the KD group received a ketogenic diet (KD: total energy content (kJ): 84% fat, 11% protein, 5% carbohydrate; C1084 from Genestil), while control group females continued receiving the CD. After weaning, offspring were transitioned to standard diets with carbohydrate content exceeding 50% (diet SAFE A04). See Table 1 for diet compositions. Pups were weekly weighed, starting at P9 until weaning.

Offspring of litter sizes <16 were randomly divided (https://www.random.org/) into two cohorts, one in France and one in Austria, and then housed under different conditions. The mice in the French cohort (n = 48; 12 animals/group, equal sex ratio) were housed in groups of 4–5 individuals per cage, grouped by sex and mixed by gestational diet. In the Austrian cohort (n = 95; KD females = 11, KD males = 31, CD females = 27, CD males = 26; 36–40 days old) the mice were housed socially, grouped by sex and within the same gestational diet group, with most animals housed with siblings (with a standard diet, with carbohydrate exceeding 50%, V1534, Ssniff, Germany).

### Glucose and BHB measurements

Glucose and BHB were measured in females during gestation (G7.5, G8.5, G10.5, G12.5 and G18.5) and 24 hours after birth and when the offspring were four months old. A superficial cut was made at the end of the tail, and 2 drops of blood were analyzed for glucose and BHB with FreeStyle Optium Neo meter (Abbott).

### Behavioral phenotyping

Behavioral phenotyping assays were conducted on 12 mice per diet and sex between three and four months of age. *Open field test (OFT):* exploratory behavior and locomotor activity were measured in open-field arenas (clear Plexiglas 40x40x40 cm^3^). Each mouse was allowed to explore a novel environment for 1 hour. The distance travelled and the time spent in the center zone (20x20 cm^2^) were recorded and analyzed using Viewpoint software (Viewpoint version 5.31.0.120). *Elevated plus maze (EPM):* anxiety-related behavior was tested in a cross-shaped maze elevated 70 cm above the floor and illuminated at 50 lux. The maze consisted of two opposing closed arms (with black Perspex walls, 20 cm high) and two open, unprotected arms. Each mouse was placed in the central platform and observed for 5 min. The time spent in the open arms, closed arms, and central platform was quantified. *Forced swim test (FST):* depression-like behavior was evaluated by placing each mouse in a transparent cylinder (height: 25 cm; diameter: 13 cm) filled with water (20 cm depth; 25°C). Each mouse was recorded for 5 min, with immobility interpreted as an indicator of depression-like behavior. *Circadian, feeding and drinking activity* were monitored simultaneously using an automated feeding/activity station (TSE Systems). Mice were placed in individual Labmaster TSE cages for 80 consecutive hours. The ambulatory and fine movement in the X dimension (total activity) was used as a parameter for spontaneous activity. Movements were detected via highly sensitive sensors embedded in the measuring platform. Data acquisition was performed on the IPNP PhenoBrain core facility.

### Reproductive success

The Austrian offspring cohort was bred at the same time, and when both groups were 6–7 months old. A total of 32 mating pairs were established, with females and males matched according to their maternal diet groups (CD group: n = 22; KD group: n = 10). Females were introduced into the male’s cage and remained together until pregnancy was visibly confirmed. The time to birth (days from pairing to delivery) was recorded, and, to measure the offspring viability, the pups were counted and sexed at weaning (P23).

### Survival studies

Survival was assessed until a humane endpoint was reached or until natural death occurred (female CD, n = 29; female KD, n = 23; male CD, n = 38; male KD, n = 43). In old mice, natural death often occurs without any warning signs of animal suffering. Throughout the study and our animal welfare assessment, the mice’s health was assessed daily, and twice a day if an animal appeared unwell; small wounds were disinfected and treated by trained personnel. Animal welfare assessment criteria included monitoring general body appearance and behavior. Mice were euthanized according to animal welfare guidelines when a humane endpoint was reached [disheveled hair, wounds, slanted eyes, abnormal posture, reduced mobility or lethargy, presence of disabling tumor (location and size) tumor-associated ascites]. Once the animals reached endpoint criteria, they were immediately euthanized by cervical dislocation. Euthanized mice (46/133) were considered non-survivors and grouped together with those that died naturally (85/133). The causes of death of n = 66 mice from the Austrian cohort (21 euthanized and 45 found death) were determined by a comprehensive pathological assessment starting at *ca*. 16 months of age. The remaining animals from the Austrian cohort and all animals from the French cohort were not submitted to pathological studies.

### Pathology

A total of 66 deceased mice from the Austrian cohort underwent comprehensive pathological assessments, including necropsy and histopathological examination. Necropsies were conducted blind to diet group allocations. During the routine pathological examination, organ samples were collected, fixed in 7% neutral buffered formalin, and embedded in paraffin wax. Tissue sections were cut to a thickness of 2.5 µm, mounted on glass slides, and stained with hematoxylin and eosin using standard protocols. When bacterial infections were suspected, microbiological testing of specific organs was performed. Parasitological examinations were routinely conducted on animals with sufficient intestinal content.

### Statistical analyses

Jamovi (version 2.3.21), SPSS (version 29.0.1.0) and R (version 4.4.2) were used for statistical analyses. Results are considered statistically significant at α < 0.05. All statistical tests are two-sided. Statistical tests and results are detailed in the figure legends and table descriptions.

## Supporting information

S1 Table Diet composition. (XLSX)

S2 Table Data for figures. (XLSX)

## References

[1] Crosby L, Davis B, Joshi S, Jardine M, Paul J, Neola M, et al. Ketogenic diets and chronic disease: weighing the benefits against the risks. Front Nutr. 2021;8:702802. doi: 10.3389/fnut.2021.702802 34336911 PMC8322232

[2] Longhitano C, Finlay S, Peachey I, Swift J-L, Fayet-Moore F, Bartle T, et al. The effects of ketogenic metabolic therapy on mental health and metabolic outcomes in schizophrenia and bipolar disorder: a randomized controlled clinical trial protocol. Front Nutr. 2024;11:1444483. doi: 10.3389/fnut.2024.1444483 39234289 PMC11371693

[3] Varaee H, Darand M, Hassanizadeh S, Hosseinzadeh M. Effect of low-carbohydrate diet on depression and anxiety: A systematic review and meta-analysis of controlled trials. J Affect Disord. 2023;325:206–14. doi: 10.1016/j.jad.2022.12.030 36584702

[4] Zhu H, Bi D, Zhang Y, Kong C, Du J, Wu X, et al. Ketogenic diet for human diseases: the underlying mechanisms and potential for clinical implementations. Signal Transduct Target Ther. 2022;7(1):11. doi: 10.1038/s41392-021-00831-w 35034957 PMC8761750

[5] Kaufman M, Nguyen C, Shetty M, Oppezzo M, Barrack M, Fredericson M. Popular dietary trends’ impact on athletic performance: a critical analysis review. Nutrients. 2023;15(16):3511. doi: 10.3390/nu15163511 37630702 PMC10460072

[6] Yang H, Shan W, Zhu F, Wu J, Wang Q. Ketone bodies in neurological diseases: focus on neuroprotection and underlying mechanisms. Front Neurol. 2019;10:585. doi: 10.3389/fneur.2019.00585 31244753 PMC6581710

[7] Puchalska P, Crawford PA. Multi-dimensional roles of ketone bodies in fuel metabolism, signaling, and therapeutics. Cell Metab. 2017;25(2):262–84. doi: 10.1016/j.cmet.2016.12.022 28178565 PMC5313038

[8] Vidali S, Aminzadeh S, Lambert B, Rutherford T, Sperl W, Kofler B, et al. Mitochondria: the ketogenic diet--A metabolism-based therapy. Int J Biochem Cell Biol. 2015;63:55–9. doi: 10.1016/j.biocel.2015.01.022 25666556

[9] Mattson MP, Moehl K, Ghena N, Schmaedick M, Cheng A. Intermittent metabolic switching, neuroplasticity and brain health. Nat Rev Neurosci. 2018;19(2):63–80. doi: 10.1038/nrn.2017.156 29321682 PMC5913738

[10] Zala D, Hinckelmann M-V, Yu H, Lyra da Cunha MM, Liot G, Cordelières FP, et al. Vesicular glycolysis provides on-board energy for fast axonal transport. Cell. 2013;152(3):479–91. doi: 10.1016/j.cell.2012.12.029 23374344

[11] Santos R, Lokmane L, Ozdemir D, Traoré C, Agesilas A, Hakibilen C, et al. Local glycolysis fuels actomyosin contraction during axonal retraction. J Cell Biol. 2023;222(12):e202206133. doi: 10.1083/jcb.202206133 37902728 PMC10616508

[12] Zala D, Schlattner U, Desvignes T, Bobe J, Roux A, Chavrier P, et al. The advantage of channeling nucleotides for very processive functions. F1000Res. 2017;6:724. doi: 10.12688/f1000research.11561.2 28663786 PMC5473427

[13] Newman JC, Verdin E. β-Hydroxybutyrate: a signaling metabolite. Annu Rev Nutr. 2017;37(1):51–76. doi: 10.1146/annurev-nutr-071816-064916 28826372 PMC6640868

[14] He Y, Cheng X, Zhou T, Li D, Peng J, Xu Y, et al. β-Hydroxybutyrate as an epigenetic modifier: underlying mechanisms and implications. Heliyon. 2023;9(11):e21098. doi: 10.1016/j.heliyon.2023.e21098 37928021 PMC10623287

[15] van der Louw EJTM, Williams TJ, Henry-Barron BJ, Olieman JF, Duvekot JJ, Vermeulen MJ, et al. Ketogenic diet therapy for epilepsy during pregnancy: A case series. Seizure. 2017;45:198–201. doi: 10.1016/j.seizure.2016.12.019 28110175

[16] Qian M, Wu N, Li L, Yu W, Ouyang H, Liu X, et al. Effect of elevated ketone body on maternal and infant outcome of pregnant women with abnormal glucose metabolism during pregnancy. DMSO. 2020;13:4581–8. doi: 10.2147/dmso.s280851 PMC7701151 33268998

[17] Whatley EG, Truong TT, Harvey AJ, Gardner DK. Acetoacetate and β-hydroxybutyrate reduce mouse embryo viability via differential metabolic and epigenetic mechanisms. Reprod Biomed Online. 2023;46(1):20–33. doi: 10.1016/j.rbmo.2022.09.018 36283935

[18] Whatley EG, Truong TT, Wilhelm D, Harvey AJ, Gardner DK. β-hydroxybutyrate reduces blastocyst viability via trophectoderm-mediated metabolic aberrations in mice. Hum Reprod. 2022;37(9):1994–2011. doi: 10.1093/humrep/deac153 35856159 PMC9433850

[19] Whatley EG, Truong TT, Harvey AJ, Gardner DK. Preimplantation embryo exposure to ketone bodies exerts sex-specific effects on mouse fetal and placental transcriptomes. Reprod Biomed Online. 2023;47(5):103320. doi: 10.1016/j.rbmo.2023.103320 37748369

[20] Whatley EG, Harvey AJ, Gardner DK. A maternal ketogenic diet alters oviduct fluid nutrients and embryo histone acetylation in mice. Reproduction. 2024;167(6):e240026. doi: 10.1530/REP-24-0026 38593828

[21] Kosiek W, Rauk Z, Szulc P, Cichy A, Rugieł M, Chwiej J, et al. Ketogenic diet impairs neurological development of neonatal rats and affects biochemical composition of maternal brains: evidence of functional recovery in pups. Brain Struct Funct. 2022;227(3):1099–113. doi: 10.1007/s00429-021-02450-1 35038032 PMC8930886

[22] Rugieł M, Setkowicz Z, Czyzycki M, Simon R, Baumbach T, Chwiej J. Element changes occurring in brain point at the white matter abnormalities in rats exposed to the ketogenic diet during prenatal life. ACS Chem Neurosci. 2024;15(21):3932–44. doi: 10.1021/acschemneuro.4c00283 39443296 PMC11587514

[23] Rugiel M, Setkowicz-Janeczko Z, Kosiek W, Rauk Z, Kawon K, Chwiej J. Does ketogenic diet used in pregnancy affect the nervous system development in offspring?─FTIR microspectroscopy study. ACS Chem Neurosci. 2023;14(15):2775–91. doi: 10.1021/acschemneuro.3c00331 37471579 PMC10401638

[24] Sussman D, Germann J, Henkelman M. Gestational ketogenic diet programs brain structure and susceptibility to depression & anxiety in the adult mouse offspring. Brain Behav. 2015;5(2):e00300. doi: 10.1002/brb3.300 25642385 PMC4309881

[25] Arqoub AMS, Flynn KG, Martinez LA. Gestational exposure to a ketogenic diet increases sociability in CD-1 mice. Behav Neurosci. 2020;134(4):358–68. doi: 10.1037/bne0000368 32223276

[26] Shaikh A, Williams DM, Stephens JW, Edwards R. Starvation ketoacidosis on the acute medical take: an easily missed complication of the keto diet. Eur J Case Rep Intern Med. 2024;11(9):004830. doi: 10.12890/2024_004830 39247239 PMC11379108

[27] Sussman D, Ellegood J, Henkelman M. A gestational ketogenic diet alters maternal metabolic status as well as offspring physiological growth and brain structure in the neonatal mouse. BMC Pregnancy Childbirth. 2013;13:198. doi: 10.1186/1471-2393-13-198 24168053 PMC4231349

[28] Parra-Vargas M, Bouret SG, Bruning JC, de Moura EG, Garland T Jr, Lisboa PC, et al. The long-lasting shadow of litter size in rodents: litter size is an underreported variable that strongly determines adult physiology. Mol Metab. 2023;71:101707. doi: 10.1016/j.molmet.2023.101707 36933618 PMC10074241

[29] Parra-Vargas M, Ramon-Krauel M, Lerin C, Jimenez-Chillaron JC. Size does matter: litter size strongly determines adult metabolism in rodents. Cell Metab. 2020;32(3):334–40. doi: 10.1016/j.cmet.2020.07.014 32814016

[30] Bogdanova OV, Kanekar S, D’Anci KE, Renshaw PF. Factors influencing behavior in the forced swim test. Physiol Behav. 2013;118:227–39. doi: 10.1016/j.physbeh.2013.05.012 23685235 PMC5609482

[31] Genzer Y, Dadon M, Burg C, Chapnik N, Froy O. Ketogenic diet delays the phase of circadian rhythms and does not affect AMP-activated protein kinase (AMPK) in mouse liver. Mol Cell Endocrinol. 2015;417:124–30. doi: 10.1016/j.mce.2015.09.012 26408964

[32] Gangitano E, Gnessi L, Lenzi A, Ray D. Chronobiology and metabolism: is ketogenic diet able to influence circadian rhythm? Front Neurosci. 2021;15:756970. doi: 10.3389/fnins.2021.756970 34819833 PMC8606558

[33] Landgraf D, Achten C, Dallmann F, Oster H. Embryonic development and maternal regulation of murine circadian clock function. Chronobiol Int. 2015;32(3):416–27. doi: 10.3109/07420528.2014.986576 25431080

[34] Seron-Ferre M, Valenzuela GJ, Torres-Farfan C. Circadian clocks during embryonic and fetal development. Birth Defects Res C Embryo Today. 2007;81(3):204–14. doi: 10.1002/bdrc.20101 17963275

[35] El-Hennamy R, Matějů K, Bendová Z, Sosniyenko S, Sumová A. Maternal control of the fetal and neonatal rat suprachiasmatic nucleus. J Biol Rhythms. 2008;23(5):435–44. doi: 10.1177/0748730408322635 18838609

[36] Orzack SH, Stubblefield JW, Akmaev VR, Colls P, Munné S, Scholl T, et al. The human sex ratio from conception to birth. Proc Natl Acad Sci U S A. 2015;112(16):E2102-11. doi: 10.1073/pnas.1416546112 25825766 PMC4413259

[37] Rivers JP, Crawford MA. Maternal nutrition and the sex ratio at birth. Nature. 1974;252(5481):297–8. doi: 10.1038/252297a0 4431446

[38] Rosenfeld CS, Grimm KM, Livingston KA, Brokman AM, Lamberson WE, Roberts RM. Striking variation in the sex ratio of pups born to mice according to whether maternal diet is high in fat or carbohydrate. Proc Natl Acad Sci U S A. 2003;100(8):4628–32. doi: 10.1073/pnas.0330808100 12672968 PMC153606

[39] Halder A, Chaudhary I, Jain M, Pandey S. Sex ratio trajectory in mouse. Reprod Biol. 2021;21(3):100514. doi: 10.1016/j.repbio.2021.100514 34049115

[40] Mao J, Zhang X, Sieli PT, Falduto MT, Torres KE, Rosenfeld CS. Contrasting effects of different maternal diets on sexually dimorphic gene expression in the murine placenta. Proc Natl Acad Sci U S A. 2010;107(12):5557–62. doi: 10.1073/pnas.1000440107 20212133 PMC2851754

[41] Gallou-Kabani C, Gabory A, Tost J, Karimi M, Mayeur S, Lesage J, et al. Sex- and diet-specific changes of imprinted gene expression and DNA methylation in mouse placenta under a high-fat diet. PLoS One. 2010;5(12):e14398. doi: 10.1371/journal.pone.0014398 21200436 PMC3006175

[42] Grabowska K, Grabowski M, Przybyła M, Pondel N, Barski JJ, Nowacka-Chmielewska M, et al. Ketogenic diet and behavior: insights from experimental studies. Front Nutr. 2024;11:1322509. doi: 10.3389/fnut.2024.1322509 38389795 PMC10881757

[43] Lindström J. Early development and fitness in birds and mammals. Trends Ecol Evol. 1999;14(9):343–8. doi: 10.1016/s0169-5347(99)01639-0 10441307

[44] Lummaa V, Clutton-Brock T. Early development, survival and reproduction in humans. Trends Ecol Evol. 2002;17(3):141–7. doi: 10.1016/s0169-5347(01)02414-4

[45] Stearns SC. The Evolution Of Life Histories. Oxford University PressOxford. 1998. doi: 10.1093/oso/9780198577416.001.0001

[46] Chandra M, Frith CH. Spontaneous neoplasms in aged CD-1 mice. Toxicol Lett. 1992;61(1):67–74. doi: 10.1016/0378-4274(92)90064-q 1609440

[47] Roberts MN, Wallace MA, Tomilov AA, Zhou Z, Marcotte GR, Tran D, et al. A ketogenic diet extends longevity and healthspan in adult mice. Cell Metab. 2017;26(3):539–546.e5. doi: 10.1016/j.cmet.2017.08.005 28877457 PMC5609489

[48] Di Meco A, Praticò D. Early-life exposure to high-fat diet influences brain health in aging mice. Aging Cell. 2019;18(6):e13040. doi: 10.1111/acel.13040 31560166 PMC6826162

[49] Zala SM, Nicolakis D, Marconi MA, Noll A, Ruf T, Balazs P, et al. Primed to vocalize: Wild-derived male house mice increase vocalization rate and diversity after a previous encounter with a female. PLoS One. 2020;15(12):e0242959. doi: 10.1371/journal.pone.0242959 33296411 PMC7725367

